# The effectiveness of acupoint herbal patching for functional dyspepsia: A protocol for systematic review and meta-analysis

**DOI:** 10.1097/MD.0000000000027682

**Published:** 2021-11-24

**Authors:** Wu Liu, Yanze Liu, Jinying Zhao, Hailin Jiang, Xiaona Liu, Lijuan Ha, Tie Li, Chengyu Liu, Fuchun Wang

**Affiliations:** a Changchun University of Chinese Medicine, China; b Graduate School, Changchun University of Chinese Medicine, China; c Department of Acupuncture, The Third Affiliated Hospital of Changchun University of Chinese Medicine, Changchun, China; d School of Rehabilitation Medicine, Changchun University of Chinese Medicine, Changchun, China; e Department of Acupuncture, The Affiliated Hospital of Changchun University of Chinese Medicine, China.

**Keywords:** acupoint herbal patching, functional dyspepsia, protocol, systematic review

## Abstract

**Background:**

Functional dyspepsia (FD) has gradually developed into a multiple disease of the digestive system that most patients may be accompanied by mental and emotional disorders, such as insomnia, anxiety, and depression. Acupoint herbal patching (AHP) is usually used as an alternative therapy for patients with FD. This study aimed to design a systematic review and meta-analysis to explore the effects of AHP on FD.

**Methods:**

We will search the Cochrane Central Register of Controlled Trials, the Web of Science, PubMed, Embase, the Chinese Biomedical Literature Database, the Chinese Scientific Journal Database, the Wan-Fang Database, and the China National Knowledge Infrastructure for randomized controlled trials of FD treated by AHP from inception to June 30, 2021. The primary outcome measures contain clinical effective rate, the symptom score of FD, and secondary outcome measures consist of quality of life, incidence of adverse events, and recurrence rate. We will use RevMan V.5.3 software to analyze data. Two reviewers will evaluate the risk of bias and the quality of the studies by the Cochrane Collaboration risk of bias tool and the Grading of Recommendations Assessment, Development, and Evaluation approach, separately.

**Results:**

This systematic review protocol will analyze the effectiveness, quality of life, improvement of the symptom, and safety of AHP therapy for FD.

**Conclusion:**

The findings of this systematic review will provide evidence to evaluate the effectiveness and safety of AHP for FD.

## 1 Introduction

Functional dyspepsia (FD) as one of the most prevalent functional gastrointestinal disease. The main performance of individuals with FD is postprandial fullness, early satiation, epigastric pain, or epigastric burning, and has no structural explanation.^[1]^ A population-based study found nearly 10% of adults in the U.S., U.K., and Canada suffer from FD based on the Rome IV standard.^[2]^ A recent global study showed that the worldwide prevalence of FD was 7.2%.^[3]^

Although a lot of pathological studies on FD has been done, its underlying pathophysiology is still unclear. The researchers proposed that there are different pathophysiological mechanisms of FD, such as gastrointestinal sensory and motor dysfunction, immune dysfunction, alterations in gastrointestinal microbiota, gut-brain axis dysfunction.^[4]^ The traditional first-line therapy of FD are proton pump inhibitors, other effective treatments include helicobacter pylori eradication treatment, phytotherapy, prokinetics, antidepressants, and psychotherapy.^[5,6]^ Because of the multifactorial nature and heterogeneity of symptoms in FD, most of existing therapies have limitations and modest effectiveness.^[4]^ Although acotiamide has been proven to improve gastric accommodation or emptying, symptoms of FD will recur about 1 year after cessation of acotiamide therapy.^[7]^ Proton pump inhibitors are the most commonly used drugs of FD, but drug abuse and adverse events have gradually increased in recent years.^[8]^ Although imipramine as a tricyclic antidepressant can treat refractory FD, these side effects of dry mouth, constipation, drowsiness, insomnia, palpitations are worth considering.^[9]^ Therefore, people who do not want to be on long-term medication are actively exploring some traditional complementary and alternative treatments for FD.^[10]^

Acupoint herbal patching (AHP) is a traditional Chinese medicine (TCM) method applied to prevent and treat various diseases, and has the characteristics of wide application and exact curative effect.^[11]^ Different studies suggest that AHP can improve clinical efficacy of digestive system diseases by adjusting gastrointestinal motility and improving clinical symptoms.^[12,13]^ Moreover, AHP can promote the recovery and reduce adverse reactions in postoperative patients.^[14,15]^ For FD, herbal patches are usually applied to CV-12, ST-36, PC-6, and ST-25 for 4 to 6 hours. However, the clinical efficacy and safety of AHP for FD still remain unclear, and need evidence-based medical research. This study aims to investigate the existing evidence of the efficacy and safety of AHP for FD, which will instruct clinicians to better use it in clinical practice.

## 2 Methods

### 2.1 Study type

We will collect relevant randomized controlled trials to evaluate clinical effectiveness, quality of life, improvement of the symptom, and safety of AHP on FD for systematic review and meta-analysis. Randomized controlled trials were conducted to compare the efficacy of AHP in the treatment of FD with no treatment, placebo, or conventional drugs, such as domperidone and mosapride. In addition, case series, observational studies and retrospective studies, qualitative studies, animal experiments, review articles will be excluded.

### 2.2 Participants

This review will include patients who had been diagnosed with FD without other limitations, such as age, gender, and race. The diagnosis of FD refers to ROME III or IV. In addition, individuals with dyspepsia were also included if they had no underlying structural or metabolic diseases for their symptoms.

### 2.3 Interventions

In the intervention group, patients received AHP as the only intervention or the main therapy combined with other interventions, such as conventional medicine, or other existing therapies of TCM. In the control group, patients were given medication, no treatment, sham or placebo, acupuncture/electro-acupuncture, and etc. The other interventions in the 2 groups should be the same.

### 2.4 Outcome measures

The primary outcomes include the following: Nepean Dyspasia Symptom Index, and overall treatment effect. The secondary outcomes include the following: Nepean Dyspepsia Life Quality Index, incidence of adverse events, and recurrence rate.

### 2.5 Search strategy

For a comprehensive literature search, we will identify relevant studies from the Cochrane Central Register of Controlled Trials, the Web of Science, PubMed, Embase, the Chinese Biomedical Literature Database, the Chinese Scientific Journal Database, the Wan-Fang Database (Wanfang), and the China National Knowledge Infrastructure from their inception onwards to June 30, 2021. The search keywords mainly includes 3 parts: intervention method, disease, and study type: (“acupoint application” or “acupoint sticker” or “herbal patch” or “herbal plaster” or “acupoint patch” or “acupoint sticking” or “point application therapy” or “plaster therapy” or “external application therapy” or “acupoint herbal patching”) and (“functional dyspepsia” or “dyspepsias” or “indigestion”) and (“randomized controlled trial” or “case-control studies”or “observational studies” or “case series” or “trial”), and (“blind”). As shown in Table 1, detailed search strategies of the PubMed Database are provided. Then, other electronic databases will use the same or adjusted search strategy. The language will be restricted to English and Chinese. In addition, the reference lists of relevant original studies will be screened to identify additional potential citations.

**Table 1:** The search strategy for PubMed database.

Number	Search terms
#1	acupoint application [MeSH]
#2	acupoint sticker [MeSH]
#3	herbal patch [MeSH]
#4	herbal plaster [MeSH]
#5	acupoint patch [MeSH]
#6	acupoint sticking [MeSH]
#7	point application therapy [MeSH]
#8	plaster therapy [MeSH]
#9	external application therapy [MeSH]
#10	acupoint herbal patching [MeSH]
#11	OR #1-#10
#12	functional dyspepsia [MeSH]
#13	dyspepsias [MeSH]
#14	indigestion [MeSH]
#15	OR #12-#14
#16	randomized controlled trial [MeSH]
#17	case-control studies [MeSH]
#18	observational studies [MeSH]
#19	case series [MeSH]
#20	trial [MeSH]
#21	OR #16-#20
#22	#11 and #15 and #21

### 2.6 Study selection and data extraction

**Figure 1:**
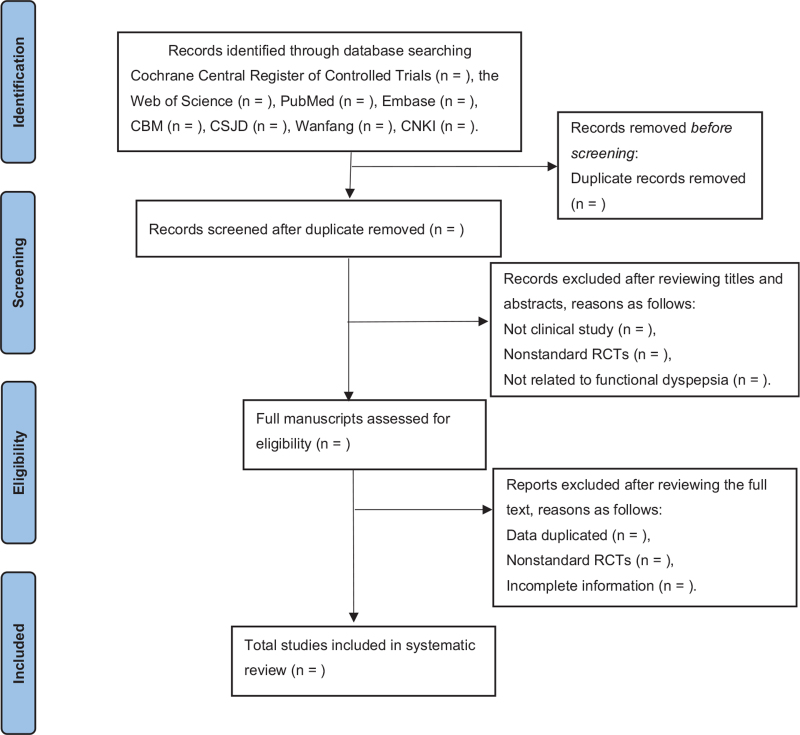
Flow chart of the search process.

In order to exclude repetitive references, we will use Note Express 3.2.0 software (http://www.inoteexpress.com/aegean/). The titles and abstracts of all citations found in the above search strategy will be independently evaluated by 2 authors (Liu W, Zhao JY). Through independent and complete reading of the full text, reviewers will include the appropriate studies. The disagreement of the 2 reviewers will be discussed with the third reviewer (Liu YZ) to reach a consensus. If it is still uncertain whether the study will be included, we will contact the author of the article to make a conclusion. At last, all data will be extracted by 2 reviewers (Liu W, Jiang HL) based on the recommendations of the Cochrane Handbook for systematic review of interventions. The following data will be extracted: author, years, written language, number of participants, duration of treatment, interventions, acupoints, herbal prescription, outcome measures, adverse events, recurrence rate, etc. The flowchart of the study selection process is shown in Figure 1.

### 2.7 Supplement of missing data

If the information in the article is incomplete, we will contact the research authors via email or phone to obtain all the information. Otherwise, we will conduct a sensitivity analysis based on the existing information to determine the possible impact of insufficient information on the results of the meta-analysis.

### 2.8 Assessment of risk of bias

Risk of bias assessment will be performed by 2 reviewers (Liu W, Liu CY) independently, using the Cochrane risk of bias tool. We will record the method of random sequence generation and allocation concealment, whether blinding was performed for participants, personnel and outcomes assessment, whether there was attrition bias of incomplete outcomes data, whether there was reporting bias of selective reporting, and whether there was other bias, such as pre-sample size estimation, early stop of trial.

### 2.9 Data synthesis and analysis

Researchers will use Review Manager 5.3 software (https://community.cochrane.org/help/tools-and-software/revman-5) to complete data analysis.^[16]^ Random or fixed effects models will be applied for meta-analysis. Continuous data will be assessed as the mean difference with a 95% confidence interval. While dichotomous data will be evaluated as risk ratio with 95% confidence interval. We will interpret it using the following criteria: I^2^ values of 25% is considered low levels of heterogeneity, 50% indicated moderate levels, and 75% indicated high levels. Since low or moderate heterogeneity suggests little variability among these studies, the data will be analyzed in a fixed-effects model. When substantial heterogeneity occurs among the studies (*P* < .05, I^2^ ≥ 50%), a random-effect model will be performed to analyze the data, and subgroup analyses will be conducted to remedy heterogeneity.

### 2.10 Additional analyses

In order to evaluate the specific impact of intervention type, age, course of disease, and course of treatment on the outcomes, we will gradually conduct subgroup analysis. Moreover, sensitivity analysis will be conducted to examine the robustness of the results. At last, we will perform complex network analysis to explore the most commonly used prescriptions for AHP to treat FD, using Spass software (Version19.0, https://www.ibm.com/analytics/spss-statistics-software).

### 2.11 Publication bias analysis

Publication bias will be assessed by funnel plots. In addition, in order to check whether the funnel plot is symmetrical, we will also use Begg test and Egger test.

### 2.12 Grading the confidence of evidence

Grading of Recommendations Assessment, Development, and Evaluation system will be used to rate the confidence in effect estimates for each outcome, and summarize the limitations in design, consistency, directness, precision, and publication bias.^[17]^ The confidence of evidence will be designated as high, moderate, low, or very low. Disagreements will be resolved by consensus.

## 3 Discussion

FD is a common and complex disease in the population, which seriously affects people's quality of life and even weight. According to the clinical symptoms, FD can be divided into postprandial distress syndrome and epigastric pain syndrome, and some patients have overlapping phenomena. Studies have found that smoking and depressive disorder are important risk factor for FD, which will increase the prevalence.^[18,19]^ FD can also cause bio-psychosocial disorder, affecting sleep quality and mood in patients.^[20]^ Therefore, refractory symptoms that do not respond to treatment should be screened for mental disorders such as anxiety, depression, and stress.^[5]^ A recent systematic review found that psychotherapy is beneficial to the treatment of FD.^[21]^

Researchers are increasingly aware of the tangible pathology that occurs in FD, such as duodenal pathology, helicobacter pylori, visceral hypersensitivity, gastrointestinal motility disorder, and others, which also represent the pathogenesis of FD.^[22]^ Studies have found that duodenal barrier disruption and immune activation play key role in the pathogenesis of FD, especially the recognition of immune activation will help the diagnosis and treatment.^[23]^ Therefore, targeted therapy based on these mechanisms provides a variety of treatment options that have modest efficacy and require further clinical research.^[24]^ On the contrary, the effect of symptom-based therapy is very limited because of the diverse pathological mechanisms of FD subtypes. At present, targeted therapy drugs include prokinetics, fundic relaxants, acid suppressive agents, neuromodulators, antidepressants, anxiolytics, digestive enzymes, histamine H1/H2 receptor antagonists, leukotriene receptor antagonists, mast cell stabilizers, and probiotics.^[25]^ Through a large number of studies, the efficacy and safety of these drugs have gradually been recognized by clinicians,^[26–29]^ and they are actively looking for alternative therapies.^[10]^

In the research of TCM treatment of FD, acupoint sticking therapy is among the most popular treatments, which is widely used for prevention and treatment of digestive diseases. Although acupoint application has the characteristics of simple operation, low cost and few side effects, there is a lack of current evidence regarding the efficacy and safety profiles of acupoint application aimed at treating FD. Therefore, this study is the first to explore the effect of acupoint application in patients with FD by systematic review and meta-analysis, and the main indicators include clinical efficacy, improvement of symptoms and quality of life, adverse events, recurrence rate, etc. Furthermore, we will analyze the acupoints and herbs used in clinical trials of acupoint application to provide a valuable references for clinicians of complementary and alternative therapies.

## 4 Ethics and dissemination

Ethics approval is not required due to this work is carried out on published data. We aimed to explore the clinical effective rate, functional outcomes, quality of life, improvement of clinical symptoms of FD, as well as effective prescriptions of AHP for patients with FD. In the end, the results will be submitted to a peer-reviewed journal.

## Author contributions

**Conceptualization:** Wu Liu, Yanze Liu.

**Data curation:** Hailin Jiang, Jinying Zhao, Xiaona Liu.

**Formal analysis:** Wu Liu, Xiaona Liu, Lijuan Ha.

**Funding acquisition:** Fuchun Wang.

**Investigation:** Jinying Zhao.

**Methodology:** Wu Liu, Chengyu Liu.

**Project administration:** Jinying Zhao, Tie Li.

**Resources:** Wu Liu, Jinying Zhao, Hailin Jiang, Chengyu Liu.

**Software:** Wu Liu, Chengyu Liu.

**Supervision:** Fuchun Wang.

**Validation:** Yanze Liu, Xiaona Liu.

**Visualization:** Fuchun Wang.

**Writing – original draft:** Wu Liu, Yanze Liu.

**Writing – review & editing:** Chengyu Liu, Tie Li, Fuchun Wang.
